# Pathology and Treatment of Jaundice

**Published:** 1862-08

**Authors:** 


### PATHOLOGY AND TREATMENT OF JAUNDICE.

Dr. George Harley read a paper on this subject (May 13, 1862) before the Royal Medical and Chirurgical Society.

It is universally admitted that the facility of the diagnasis of jaundice is only equalled by the obscurity of its pathology and the uncertainty of its treatment. In this communication, therefore, the author set about unravelling the nature of the various morbid conditions which give rise to it; and pointed out how, notwithstanding the seeming discord, they could all appropriately come under the two common heads of "jaundice from suppression of the biliary function," and "jaundice from the reabsorption of the secreted but retained bile." Moreover, Dr. Harley showed that the pathology of jaundice resulting from suppression is totally different from that arising from obstruction: and, consequently, that a line of treatment which would be appropriate and beneficial in the one form, would be detrimental, if not actually hazardous, in the other. Fortunately, however, the author pointed out a new method of distinguishing the two forms of the disease when all the ordinary means of symptomatic and physical diagnosis prove unavailing. The method consists in analyzing the urine, which, he finds, contains different morbid products according to the particular form of the disease. Thus, tor example, in jaundice from suppression the urine contains only those biliary ingredients which exist performed in the blood. In jaundice from obstruction, on the other hand, the urine contains, in addition to these, the materials generated in the liver itself, and which have been reabsorbed into the circulation from the distended gall-bladder and ducts. A simple mode of distinguishing the two conditions is, to add to about two drachms of urine half a drachm of strong sulphuric acid, and a fragment of loaf sugar the size of a pea. If at the line of contact of the two liquids a scarlet or purple color is produced, it proves that the acids of the bile are present, and the case may consequently be put down as one of jaundice from obstruction. On the other hand, if no bile-acid reaction, but merely a browning of the sugar, be observed, the case is in all probability one of suppression. Dr. Harley pointed out, however, that care must be taken not to confound the two cases; as jaundice from obstruction, especially the severe form, often merges into jaundice from suppression. The author also confirmed Frerich's statement regarding the presence of tyroeine and leucine in the urine of acute atrophy of the liver; and further stated that he had also found these substances in the urine of chronic atrophy, so that their presence might aid in the diagnosis of the latter as well as of the former condition of the hepatic organ. Several cases were cited illustrating the value of the different methods of diagnosis; and the author concluded by pointing out the class of cases in which mercury and other remedies were likely to be beneficial, and where they were likely to do injury. He specially recommended the employment of benzoic acid in jaundice from suppression, and inspissated bile in that arising from obstruction, in which latter class the patient frequently dies from slow starvation, in consequence of the absence of bile in the digestive process causing imperfect assimilation of the food to take place. Dr. Harley also called special attention to the fact that bile, as now employed, more frequently does harm than good; for, when given along with the food, instead of aiding the digestive process, it actually retards it by interfering with the action of the gastric juice. If, on the other hand, bile be administered, as the author proposes, at the end of the stomachal digestion, it acts (as in the healthy organism) on the chyme, and renders it fit for absorption. In order still further to insure this desirable object, Dr. H. has had bile specially prepared and put up into capsules, which are not readily acted on by the gastric juice, but which, on being dissolved in the duodenum, allow the bile to come in contact with the food at the proper moment, and thereby enable the physician to imitate nature, and supply an important element to the system. The communication was well illustrated with preparations and drawings.—*Med. Times & Gaz.*

